# Real-World Practice of Hypofractionated Radiotherapy in Patients With Invasive Breast Cancer

**DOI:** 10.3389/fonc.2022.811794

**Published:** 2022-02-03

**Authors:** Fang Chen, Timothy S.K. Hui, Lingyu Ma, Yaqing Nong, Ying Han, Haiman Jing, Eric K.W. Lee, Zhiyuan Xu, Pingfu Fu, Amy Tien Yee Chang, Victor Hsue, Feng-Ming Spring Kong

**Affiliations:** ^1^ Department of Clinical Oncology, The University of Hong Kong-Shenzhen Hospital, Shenzhen, China; ^2^ Department of Clinical Oncology, Li Ka Shing Faculty of Medicine, The University of Hong Kong, Hong Kong, Hong Kong SAR, China; ^3^ Department of Population and Quantitative Health Sciences, Case Western Reserve University, Cleveland, OH, United States; ^4^ Comprehensive Oncology Center, Hong Kong Sanatorium & Hospital, Hong Kong, Hong Kong SAR, China

**Keywords:** breast cancer, hypofractionated radiotherapy, conventionally fractionated radiotherapy, real-world practice, molecular subtype

## Abstract

**Purpose:**

Application of hypofractionated radiotherapy (HFRT) is growing in patients with breast cancer (BC). This study aimed to explore a real-world practice of HFRT in early and locally advanced BC.

**Methods:**

Patients with invasive BC between 2015 and 2019 were retrospectively reviewed. Radiotherapy (RT) was delivered by HFRT and conventionally fractionated radiotherapy (CFRT). Locoregional recurrence-free survival (LRRFS) and disease-free survival (DFS) were calculated by Kaplan–Meier curve and compared by Log-rank test. The effect of treatment modality on DFS was estimated by univariate and multivariable analyses.

**Results:**

A total of 1,010 patients were included in this study, and 903 (89.4%) were treated with HFRT. At a median follow-up of 49.5 months, there was no significant difference in a 4-year cumulative incidence of LRRFS in HFRT group (1.5%) and in CFRT group (3.8%) (p = 0.23), neither in different nodal stages nor in N2–3 patients with different molecular subtypes. The 4-year DFS was 93.5% in HFRT group compared with 89.9% in CFRT group with no significant difference either (p = 0.17). Univariate and multivariable analyses also showed no significant difference in DFS between HFRT and CFRT group. However, DFS of HFRT group tended to be lower in N2–3 patients with triple negative BC compared with that of CFRT group (76.2% versus 100%).

**Conclusion:**

HFRT can achieve similar cumulative incidence of LRRFS and DFS in patients with BC after lumpectomy or mastectomy, and also in different nodal stage, and in locally advanced stage with different molecular subtypes.

## Introduction

Adjuvant Radiotherapy (RT) can reduce local recurrence and improve survivals for patients with breast cancer (BC) (1–5). Based on randomized trials (6, 7), the international standard of RT for BC patients after mastectomy or lumpectomy is conventionally fractionated radiotherapy (CFRT) of 50 Gy in 25 fractions over 5 weeks.

In recent years, the application of hypofractionated radiotherapy (HFRT) has been growing, due to its shorter overall treatment course, with similar local control and survival benefits (8). Several prospective trials demonstrated that HFRT had similar disease control and toxicity compared with CFRT in patients with early stage BC after breast-conserving therapy (BCT) (9–15). As for regional lymph nodal positive cases, START Trials A and B also showed HFRT group had similar locoregional tumor control and late normal tissue effects in patients with pT1-3aN0-1 BC (13–15). In the meantime, it should be noted that these two trials had not enrolled patients with locally advanced BC (e.g., T4 or N2–3) and the proportion of patients who received mastectomy was relatively low (8–15%) (15). As for patients with locally advanced BC (pT3-4N2–3), post-mastectomy HFRT was reported to have non-inferiority of a 5-year cumulative incidence of LRR and similar toxicities to CFRT (16). However, none of the patients in this trial included irradiation of internal mammary nodal regions (IMN) which was applied as part of locoregional treatment in several studies (1, 2, 4) and is recommended by the ASCO guideline (17); the vast majority (96%) of patients underwent two-dimensional RT with less than 2% patients receiving advanced RT technology of intensity-modulated radiotherapy (IMRT).

Since evidence of HFRT in patients with locally advanced BC is not sufficient, real-world practice varies from institution to institution (18), and survival outcomes from large study population in the real world are urgently needed. Meanwhile, tumor characteristics and survivals vary in different molecular subtypes. The MA.20 trial found that a 10-year overall survival (OS) of nodal-irradiation group was significantly higher than that of control group in ER-negative tumors (1), suggesting the possibility of individualized RT strategy in patients with different molecular subtypes. However, the remaining prospective randomized studies rarely address this. Therefore, real-world practice also varies in physicians and they may choose different RT modalities based on molecular subtypes. Studies of HFRT in patients with detailed subtypes are needed to guide clinical decision, especially in aggressive triple negative breast cancer (TNBC).

Shenzhen is a young and economically developing city in southern China. However, the medical resources were relatively insufficient in the last few years with only three linear accelerators operating in the city during 2013 to 2019, while the permanent resident population was 13.4388 million in 2019. The average waiting time of getting RT in our institution was as long as 2–3 months during that time. Most BC patients were thus offered HFRT to maximize the use of medical resources. This study aimed to retrospectively explore the survivals of patients who had received HFRT and CFRT in a single institution.

## Methods

### Study Population

Patients with invasive BC who received adjuvant RT between January 2015 and May 2019 in the University of Hong Kong-Shenzhen Hospital formed the initial cohort of this retrospective study. Patients with locoregional recurrent tumor or metastatic disease at diagnosis, breast lymphoma or breast phyllodes tumor, or with other malignant tumors at the time of RT were excluded. Patients in this study underwent surgery and systemic therapy in various hospitals in this southern city of China but received RT in our same institution.

### Procedures

HFRT was delivered at 40.05 Gy in 15 fractions, once daily, five times per week, over 3 weeks. CFRT was delivered at 50 Gy in 25 fractions, once daily, five times per week, over 5 weeks. Patients who had an immediate surgical reconstruction, or with supraclavicular lymph nodes and/or IMN involvement were still offered with CFRT alone or with simultaneous integrated boost to a total of 60 or 62.5 Gy in 25 fractions to the involved lymph node respectively. Other patients were usually offered with HFRT.

Radiotherapy techniques employed included 2-field tangential opposing technique (2-field), tangential opposing fields with an anterior supraclavicular fossa (SCF) field (three-dimensional conformal technique, 3DCRT), and volume modulated arc therapy (VAMT). The 2-field technique was normally employed on patients who needed irradiation of only the breast for BCT. 3DCRT technique was usually employed on patients who needed irradiation of the breast or chest wall and SCF and/or axillary fossa. Generally, full axillary fossa irradiation was not recommended except for patients with nodal involvement but inadequate axillary lymph node dissection (ALND) or patients with extensive bulky nodal involvement. The indications of IMN irradiation in our institution were invasive BC with all N3 diseases or N2 diseases with centrally or medially located primary tumors. VMAT composed of 2 partial arcs was normally employed on patients who needed irradiation of the IMN, together with the irradiation of breast or chest wall and SCF, level III axillary nodal region.

Most patients were treated with 6 MV photons. For patients who underwent breast-conserving surgery, 6–12 Mev electron boost of 10–16 Gy in 5–8 fractions was delivered to tumor bed after whole breast irradiation, depending on resection margin. The use of bolus over chest wall for patients with mastectomy was applied according to tumor invasion and surgical resection margins. Deep inspiration breath-hold technique was used to reduce mean heart doses for patients with unacceptably high heart doses if treated without breathing control.

### Statistical Analysis

The primary endpoints were locoregional recurrence-free survival (LRRFS) and disease-free survival (DFS). LRR was defined as an invasive disease recurrence in the ipsilateral chest wall or breast, or regional lymph nodes (ipsilateral axillary, internal mammary or supraclavicular fossa nodes) within the irradiated target volume. LRRFS was defined as the interval from the date of surgery to the date of having LRR and was censored at last follow-up date for those without LRR. DFS was defined as the interval from the date of surgery to the first documented occurrence of an event defined as invasive locoregional recurrence, distant recurrence, or death from any cause. Patients alive without an event as of the analysis cutoff date were censored at last follow-up date. Patients with a follow-up duration less than 6 months were excluded from the analysis for survivals.

The difference of continuous patient characteristic variables between the two cohorts of patients with HFRT or CFRT was made using a T-test and the association between two categorical factors was examined using Chi-square tests. The probability of LRRFS and DFS was estimated by the Kaplan–Meier method, and their difference between groups was compared by Log-rank test. The effect of treatment modality (HFRT versus CFRT) on DFS was evaluated by univariate Cox analysis and further estimated using multivariable Cox model controlling the effects of tumor stage, subtypes, chemotherapy strategy, RT technology and RT fields with effect size of predictors estimated using hazard ratio (HR) and corresponding 95% confidence interval. Variables (p >0.1) were excluded in the multivariable Cox regression unless they held appreciable clinical significance. Meanwhile, the collinearity testing was performed using the variance inflation factor (VIF), and VIF >4.0 was interpreted as indicating multicollinearity. Variables with VIF >4.0 were not included in the final Cox model. Harrell’s C-statistic was calculated by 1,000-fold bootstrap resampling iterations to an initial fitted Cox model in the derivation set. Likelihood ratio tests of treatment according to covariate interactions were used to examine the heterogeneity of the treatment effect according to subgroups of age, T stage, N stage, subtypes, chemotherapy strategy, RT technology and RT fields. Statistical analysis was performed using R software (version 3.6.1; https://www.R-project.org). All P-values were two-sided and statistical significance was set at P <0.05.

## Results

### Study Population

**Figure 1:**
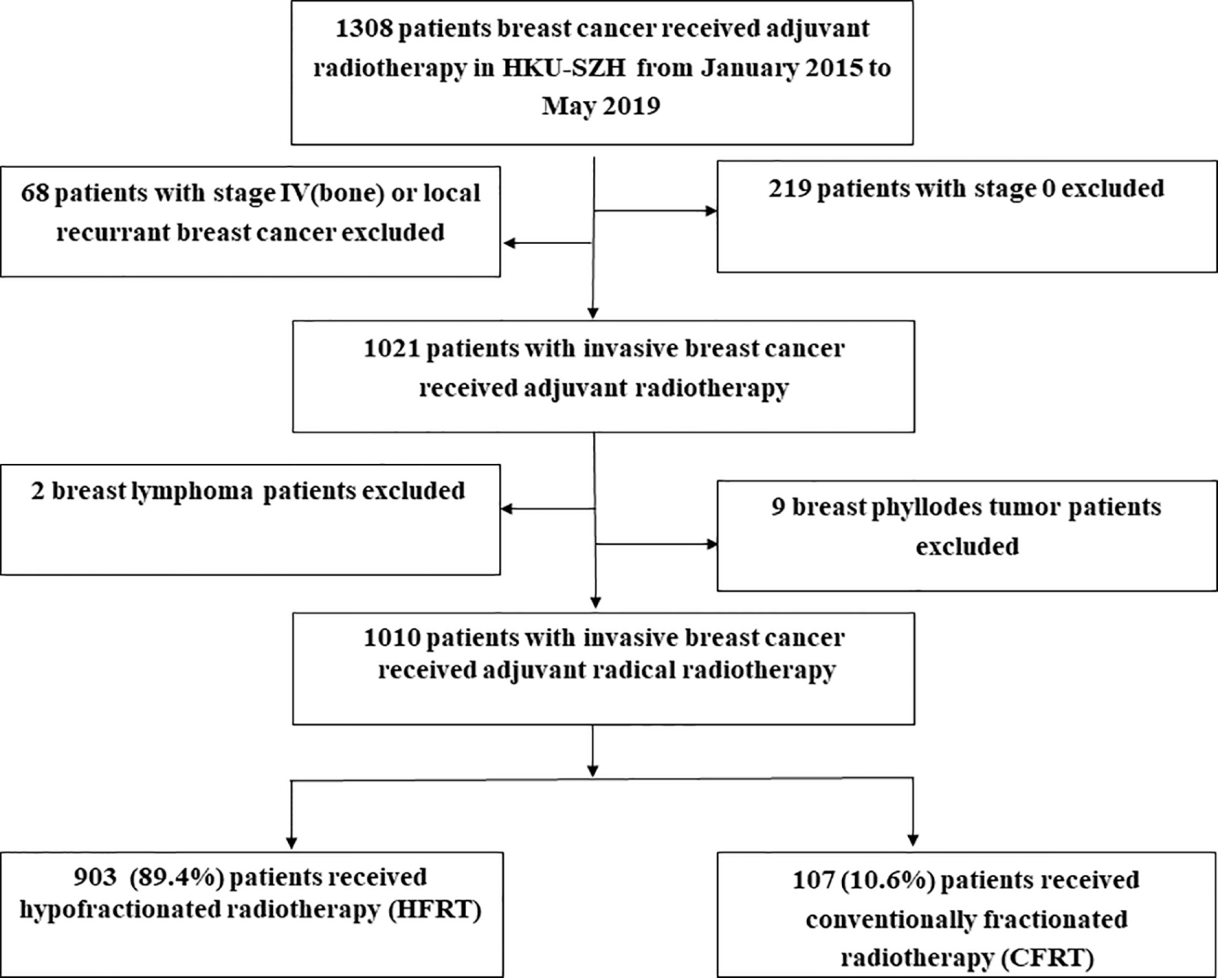
Study population. As shown, 903 (89.4%) patients received hypofractionated radiotherapy (HFRT) and 107 (10.6%) patients were treated with conventional fractionated radiotherapy (CFRT).

During January 2015 to May 2019, a total of 1,010 patients with invasive BC were included in this study. The cut-off date for data collection was August 15, 2021. Among them, 903 (89.4%) patients had received HFRT and 107 (10.6%) patients were treated with CFRT (**Figure 1**). **Table 1** lists the baseline characteristics of the patient, tumor and treatment factors. A total of 185 (18.3%) patients received neoadjuvant chemotherapy (NACT) and we used modified stage in this study because RT was offered according to the higher stage for patients receiving NACT. Modified tumor (T), nodal (N) stage were identified as the higher T, N stage between clinical T, N and pathological T, N stage for patients who had received NACT and were identified as pathological T, N stage for patients who had upfront surgery. **Supplementary Table 1** lists the detailed radiation dosimetric factors of these two groups.

**Table 1:** Baseline characteristics.

	Hypofractionated radiotherapy (HFRT) (n = 903)	Conventionally fractionated radiotherapy (CFRT) (n = 107)	p-value
Age			<0.05
Median (range)—year	47 (26, 86)	42 (24, 78)	
Menopausal status—no. (%)			0.01
Premenopausal	656 (72.6%)	90 (84.1%)	
postmenopausal	247 (27.4%)	17 (15.9%)	
Tumor laterality—no. (%)			0.38
left	444 (49.2%)	60 (56.1%)	
right	458 (50.7%)	47 (43.9%)	
bilateral	1 (0.1%)	0 (0%)	
Modified T stage—no. (%)			0.08
T1	440 (48.7%)	32 (29.9%)	
T2	390 (43.2%)	49 (45.8%)	
T3	49 (5.4%)	17 (15.9%)	
T4	24 (2.7%)	9 (8.4%)	
Modified N stage—no. (%)			<0.05
N0	364 (40.3%)	13 (12.1%)	
N1	328 (36.3%)	43 (40.2%)	
N2	135 (15.0%)	22 (20.6%)	
N3	76 (8.4%)	29 (27.1%)	
Modified stage—no. (%)			<0.05
I (IA/IB)	246 (27.2%)	6 (5.6%)	
II (IIA/IIB)	419 (46.4%)	41 (38.3%)	
III (IIIA/IIIB/IIIC)	238 (26.4%)	60 (56.1%)	
ER positive—no. (%)	682 (75.5%)	72 (67.3%)	0.08
PR positive—no. (%)	604 (66.9%)	70 (65.4%)	0.84
HER2 positive—no. (%)	250 (27.7%)	31 (29.0%)	0.87
Subgroups—no. (%)			0.28
HR^+^/HER2^−^	539 (59.7%)	56 (52.3%)	
HER2^+^/HR^−^	101 (11.2%)	11 (10.3%)	
HER2^+^/HR^+^	149 (16.5%)	20 (18.7%)	
HR^−^/HER2^−^	114 (12.6%)	20 (18.7%)	
Breast surgery procedure—no. (%)			<0.05
Breast conserving therapy (BCT)	470 (52.0)	7 (6.5%)	
Mastectomy	433 (48.0%)	100 (93.5%)	
Axillary nodes procedure—no. (%)			<0.05
Sentinel lymph node biopsy only	353 (39.1%)	8 (7.5%)	
Axillary lymph node dissection (ALND)	550 (60.9%)	99 (92.5%)	
Close or positive margin—no. (%)	47 (5.2%)	5 (4.7%)	1.00
Chemotherapy—no. (%)	812 (89.9%)	104 (97.2%)	<0.05
Chemotherapy strategy— no. (%)			<0.05
None	91 (10.1%)	3 (2.8%)	
Neoadjuvant	124 (13.7%)	34 (31.8%)	
Adjuvant	665 (73.6%)	66 (61.7%)	
Neoadjuvant + adjuvant	23 (2.6%)	4 (3.7%)	
Anti-HER2 target therapy—no. (%)	236 (26.1%)	30 (28.0%)	0.58
Endocrine therapy—no. (%)	688 (76.2%)	76 (71.0%)	0.29
Radiotherapy technique—no. (%)			<0.05
VMAT	130 (14.4%)	34 (31.8%)	
2-field	348 (38.5%)	5 (4.7%)	
3DCRT	425 (47.1%)	68 (63.5%)	
RT fields—no. (%)			<0.05
Breast alone	350 (38.8%)	5 (4.7%)	
Breast/chest wall + SCF	411 (45.5%)	56 (52.3%)	
Breast/chest wall + SCF + IMN	137 (15.2%)	44 (41.1%)	
Breast/chest wall + SCF + Axillary	5 (0.5%)	2 (1.9%)	
RT Dose and fractions—no. (%)			<0.05
40.5 Gy/15 fx	903 (100%)	0 (0%)	
50 Gy/25 fx	0 (0%)	95 (88.8%)	
60–62.5 Gy/25–26 fx	0 (0%)	12 (11.2%)	
Electron boost			<0.05
10 Gy/5 frs	437 (48.4%)	8 (7.5%)	
16 Gy/8 frs	51 (5.6%)	5 (4.7%)	
Use of RPM—no. (%)	13 (1.4%)	8 (7.5%)	<0.05

T, Tumor; N, Nodal; ER, estrogen receptor; PR, progesterone receptor; HR, hormone receptor; HER2, human epidermal growth factor receptor 2; 2D-fields, 2-field tangential opposing technique; 3DCRT, three-dimensional conformal technique; VMAT, volume modulated arc therapy; SCF, supraclavicular lymph nodes; IMN, internal mammary nodal; RT, radiotherapy; fx, fractions; RPM, real-time position management.

### Treatment Outcomes

**Figure 2:**
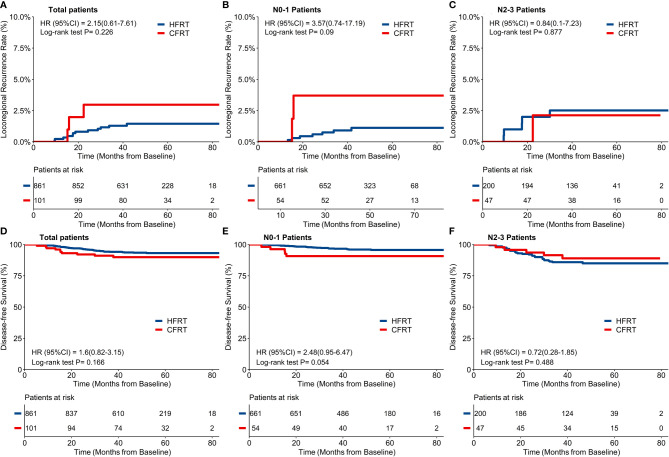
Locoregional recurrence-free survival (LRRFS) and disease-free survival (DFS). There was no significant difference of 4-year cumulative incidence of LRRFS between hypofractionated radiotherapy (HFRT) and conventional fractionated radiotherapy (CFRT) group (1.5% and 3.8% respectively, p = 0.23, **(A)**, neither in patients with N0-1 stage **(B)** nor N2-3 stage **(C)**. HFRT achieved similar DFS compared with CFRT (93.5% and 89.9% respectively, p = 0.17, **(D)**, and also in patients with N0-1 stage **(E)** and N2-3 stage **(F)**.

Forty-eight (4.8%) patients were lost for follow-up and the remaining 962 patients were included in the final analysis with a median follow-up time of 49.5 months (IQR: 40.5–61.9). By the time of the last follow-up date, 15/962 patients had developed LRR (12/861 in HFRT group and 3/101 in CFRT group). Twelve patients had local recurrence in breast or chest wall (11/861 in HFRT group and 1/101 in CFRT group), and 5 patients had regional recurrence in lymph nodes (2/861 in HFRT group and 3/101 in CFRT group). As shown in **Figure 2A**, a 4-year cumulative rate of LRRFS was 1.5% (95%CI: 0.9–2.6%) in HFRT group compared with 3.8% (95%CI: 1.0–8.9%) in CFRT group (HR 2.15, 95%CI 0.61–7.61, p = 0.23). There were no significant differences of 4-year cumulative incidence of LRRFS in patients with either N0–1 or N2–3 disease (**Figures 2B, C**).

By the time of the last follow-up date, 65/962 patients had developed disease recurrence (55/861 in HFRT group and 10/101 in CFRT group), at a rate higher than that of the locoregional failure in this study. Sixteen (1.7%) patients died (12/861 in HFRT group and 4/101 in CFRT group) with 14 patients who died of BC (10/861 in HFRT group and 4/101 in CFRT group). As shown in **Figure 2D**, 4-year DFS of 962 patients was 93.5% (95%CI: 91.8–95.2%) in HFRT group compared with 89.9% (95% *CI: 84.2–96.1%) in CFRT group (HR 1.60, 95%CI 0.82–3.15, p = 0.17). There were no significant differences of 4-year DFS in patients with either N–1 or N2–3 diseases (**Figures 2E, F**).

**Figure 3:**
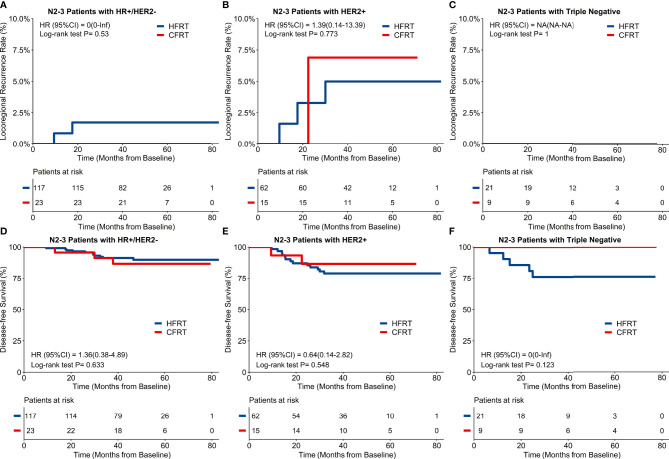
Locoregional recurrence-free survival (LRRFS) and disease-free survival (DFS) and molecular subtypes in 247 patients with locally advanced N2-3 breast cancer. There were no significant differences of 4-year cumulative incidence of LRRFS between hypofractionated radiotherapy (HFRT) and conventional fractionated radiotherapy (CFRT) group with HR+/HER2- **(A)**, HER2+ **(B)** and triple negative breast cancer (TNBC) **(C)**. DFS between HFRT and CFRT group was similar in N2-3 patients with HR+/HER2- **(D)** and HER2+ **(E)** breast cancer with no significant difference (ps > 0.05), while N2-3 patients with TNBC had numerically lower DFS in HFRT group than in CFRT group (76.2% and 100% respectively, **F**).

For the 247 patients with locally advanced N2–3 BC, a 4-year cumulative incidence of LRRFS was 2.5% (95%CI: 1.1–5.9%) in HFRT group compared with 2.1% (95%CI: 0.3–14.2%) in CFRT group (HR 0.84, 95%CI 0.10–7.23, p = 0.88) (**Figure 2C**). As shown in **Figure 2F**, 4-year DFS was 85.0% (95%CI: 80.1–90.3%) in HFRT group compared with 89.0% (95%CI: 80.4–98.6%) in CFRT group in patients with N2–3 BC (HR 0.72, 95%CI 0.28–1.85, p = 0.49). LRRFS and DFS between HFRT and CFRT group in N2–3 patients were further investigated in groups according to subtypes (**Figure 3**). As shown in **Figure 3F**, 4-year DFS in locally advanced N2–3 patients with TNBC was numerically lower in HFRT group (76.2%) than in CFRT group (100%) while 4-year cumulative incidence of LRRFS was 0% in both groups (**Figure 3C**) (p = 0.12). DFS between HFRT and CFRT group was similar in N2–3 patients with subtype of HR^+^/HER2^−^ and HER2^+^, with no significant difference either (ps >0.05) (**Figures 3D, E**).

Univariate analysis showed DFS was significantly correlated with the primary T stage, regional N stage, stage, ER status, PR status, HER2 status, subtypes, breast surgery procedure, radiotherapy technique and RT fields, but not the RT fractionation of CFRT or HFRT, age, menopausal status, surgical margin, and chemotherapy strategy (**Table 2**). Some highly correlated variables such as stage, ER status, PR status, HER2 status, radiotherapy technique and RT fields were not included in the final multivariable analyses (C-index 0.75; 95% CI: 0.70-0.81, bootstrap optimism-corrected C-indexes: 0.72). Further multivariable analyses showed DFS was significantly associated with primary tumor stage, regional node stage and subtypes, but not RT fractionation of CFRT or HFRT and breast surgery procedure. Patients with locally advanced diseases (T3–4 or N2–3), and aggressive subtypes of HER2^+^/HR^−^ and triple negative had significantly poorer DFS. RT fractionation of CFRT or HFRT was not one of the significant factors for DFS (p = 0.53).

**Table 2:** Univariate and multivariable Cox analyses of disease-free survival (DFS).

		Univariate		multivariable	
	n (%)	HR	p-value	HR	p-value
RT fractionation					
CFRT	861 (89.5%)	1		1	
HFRT	101 (10.5%)	1.60 (0.82, 3.15)	0.17	0.79 (0.38, 1.64)	0.53
Age					
45 y	494 (51.4%)	1			
>45 y	468 (48.6%)	1.00 (0.98, 1.03)	0.83		
Menopausal status					
Premenopausal	711 (73.9%)	1			
postmenopausal	251 (26.1%)	1.09 (0.63, 1.88)	0.75		
Modified T stage					
T1–2	868 (90.2%)	1		1	
T3–4	94 (9.8%)	4.06 (2.36, 6.99)	<0.05	2.50 (1.38, 4.54)	<0.05
Modified N stage					
N0	360 (37.4%)	1		1	
N1	355 (36.9%)	1.87 (0.90, 3.90)	0.10	1.54 (0.65, 3.62)	0.32
N2	147 (15.3%)	3.77 (1.75, 8.12)	<0.05	2.95 (1.21, 7.21)	<0.05
N3	100 (10.4%)	6.36 (3.00, 13.47)	<0.05	4.70 (1.91, 11.59)	<0.05
Modified N stage					
N0–1	715 (74.3%)	1			
N2–3	247 (25.7%)	3.36 (2.07, 5.47)	<0.05		
Modified stage					
I	245 (25.5%)	1			
II	433 (45.0%)	3.58 (1.25, 10.29)	<0.05		
III	284 (29.5%)	8.33 (2.96, 23.40)	<0.05		
ER status					
Negative	244 (25.4%)	1			
Positive	718 (74.6%)	0.35 (0.22, 0.58)	<0.05		
PR status					
Negative	318 (33.1%)	1			
Positive	644 (66.9%)	0.43 (0.27, 0.70)	<0.05		
HER2 status					
Negative	695 (72.2%)	1			
Positive	267 (27.8%)	1.67 (1.02, 2.76)	<0.05		
Subtypes					
HR^+^/HER2^−^	569 (59.1%)	1		1	
HER2^+^/HR^−^	108 (11.2%)	3.58 (1.95, 6.57)	<0.05	2.66 (1.43, 4.94)	<0.05
HER2^+^/HR^+^	159 (16.5%)	1.07 (0.49, 2.35)	0.87	0.95 (0.43, 2.10)	0.90
HR^−^/HER2^−^	126 (13.1%)	2.25 (1.16, 4.35)	<0.05	2.32 (1.18, 4.55)	<0.05
Breast surgery procedure					
Breast conserving therapy	507 (5.7%)	1		1	
Mastectomy	455 (47.3)	0.38 (0.22, 0.66)	<0.05	0.86 (0.41, 1.78)	0.68
Margin					
Negative	914 (95.0%)	1			
Close or positive	48 (5.0%)	0.93 (0.29, 2.95)	0.90		
Chemotherapy strategy					
None	92 (9.6%)	1			
Neoadjuvant	180 (18.7%)	0.85 (0.37, 2.07)	0.71		
Adjuvant	690 (71.7%)	0.67 (0.34, 1.50)	0.30		
Radiotherapy technique					
VAMT	155 (16.1%)	1			
2-field	336 (34.9%)	0.15 (0.07, 0.32)	<0.05		
3DCRT	471 (49.0%)	0.38 (0.22, 0.64)	<0.05		
Electron boost					
None	484 (50.3%)	1			
Yes	478 (49.7%)	0.37 (0.22,0.64)	<0.05		
RT fields					
Tangential breast only	338 (35.1%)	1			
Breast/chest wall + SCF	453 (47.1%)	2.27 (1.07, 4.83)	<0.05		
Breast/chest wall + SCF + IMN	171 (17.8%)	6.96 (3.29, 14.69)	<0.05		

RT, radiotherapy; HFRT, hypofractionated radiotherapy; CFRT, conventionally fractionated radiotherapy; T, Tumor; N, Nodal; ER, estrogen receptor; PR, progesterone receptor; HR, hormone receptor; HER2, human epidermal growth factor receptor 2; 2-field, 2-field tangential opposing technique; 3DCRT, three-dimensional conformal technique; VAMT, volume modulated arc therapy; SCF, supraclavicular lymph nodes; IMN, internal mammary nodal.

**Figure 4:**
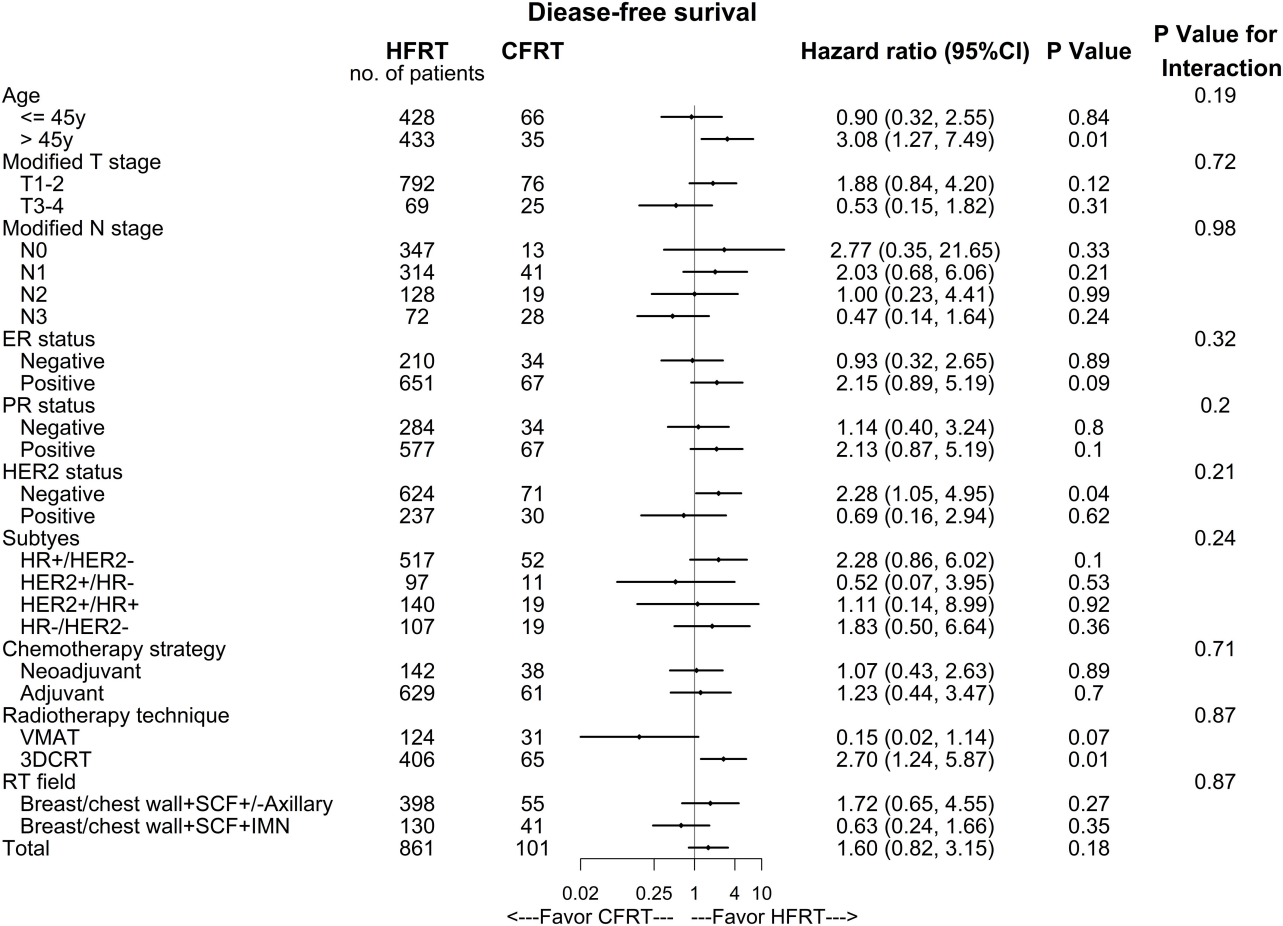
Disease-free Survival (DFS) of subgroup analyses in hypofractionated radiotherapy (HFRT) and conventionally fractionated radiotherapy (CFRT) group. DFS was better in HFRT group for patients with age >45 years, HER2-negative or receiving 3DCRT, while there were no significant differences in most of other clinic factors.

In subgroup analyses (**Figure 4**), the treatment benefits of DFS seems to be better in HFRT group for patients with age >45 years, HER2-negative or receiving 3DCRT, while there were no significant differences in most of other clinical factors such as T stage, N stage, and ER status.

## Discussion

This real-world study demonstrated that HFRT can achieve similar cumulative incidence of LRRFS in 1,010 patients with BC compared with CFRT, and also in various nodal stages and in locally advanced stage with different molecular subtypes. There were no significant differences of DFS between HFRT and CFRT group either. However, locally advanced patients with TNBC tended to have lower DFS in HFRT group than in CFRT group while the cumulative incidence of LRRFS was similar. Univariate and multivariable analyses also showed RT fractionation of CFRT or HFRT was not a significant risk factor for DFS in patients with BC.

It is encouraging to note that HFRT generated similar tumor control outcomes as the CFRT in this real-world study from China. Our results were consistent with multiple non-inferiority trials on HFRT in patients with early stage BC after BCT (9–15), in high risk patients after mastectomy (16) and real-world studies from Thailand and India (19, 20). These results are supported by the radiobiological rationale that BC cells are considered to be more susceptible to larger radiation dose per fraction compared to conventional fraction size (21). CFRT is based on a historical assumption that BC cells are less sensitive to the changes in the radiation dose per fraction than the adjacent normal tissues. However, BC might be more sensitive to changes in the radiation dose per fraction compared with most of other cancers (22).

The second rationale of HFRT no inferiority could be the improvement of overall long-term survival from systemic therapy during last couple of decades and the role of RT to improve survival by locoregional control might be partially offset. The two randomized trials supported CFRT recruited patients 30 years ago (1982 to 1989) with outdated systemic therapy of cyclophosphamide, methotrexate, and fluorouracil (CMF) or tamoxifen which had been replaced by more effective systemic regimens years ago (6, 7). The LRR, DFS and OS were 9, 48, and 54% at 10 years in patients receiving radiotherapy plus CMF in Trial 82b (6), and 8, 36, and 45% at 10 years in patients receiving radiotherapy plus tamoxifen in Trial 82c, respectively (7). Nowadays, chances of survival are improved remarkably by advances in systemic therapy, especially for patients with hormone-receptor (HR) positive and HER2-positive BC. The DFS and OS could be as high as 83.1 and 87.2% at 8 years for patients with HR positive BC who received adjuvant chemotherapy and endocrine therapy of exemestane plus ovarian suppression (23). The invasive-disease-free survival and OS could also be 91 and 95% at 6 years for HER2-positive patients who received adjuvant chemotherapy with concurrent pertuzumab and trastuzumab (24). In the meanwhile, local recurrence could be reduced by systemic treatment (25). Wang et al. reported trastuzumab significantly reduced the 5-year LRR in HER2-positive patients after RT (26). In another *post hoc* study, trastuzumab was also reported to reduce LRR and improve DFS in patients with locally advanced HER2-positive BC receiving RT (27). Therefore, HFRT could be considered in BC patients with improved long-term survivals and reduced local failure by concurrent systemic treatment. In a large analysis of 15779 patients with HER2-positive BC, the 5-year OS rate was similar between HFRT and CFRT groups (93.9% versus 95.2%, p = 0.26) with concurrent trastuzumab (28). In fact, the challenge for patients with BC is distant metastasis which more relies on systemic treatment. Nerveless, the non-inferior DFS of HFRT in this real world study suggests the appropriateness of such treatment in various stages of BC.

In addition, the adverse effects appear to be similar, or even lower in HFRT group (14, 29). It is known from the START Trials that breast shrinkage, telangiectasia, and breast edema were significantly less common in HFRT group than in CFRT group at follow-up of 10 years (15). In patients with locally advanced BC post mastectomy, HFRT also had similar late complications (e.g., lymphoedema, shoulder dysfunction, and ischemic heart disease) and less frequent grade 3 acute skin toxicity than CFRT (16). In the meanwhile, advanced RT techniques such as VAMT are employed nowadays to have better protection for the organs at risk. Radiation pneumonitis might be reduced by more conformal radiation technique such as VMAT, especially in patients who need irradiation of regional nodes (30). Thus, HFRT can be safely applied in patients with BC with similar toxicities, or even lower toxicities with improved RT techniques.

With the above rationales and evidence from various clinical trials, one may conclude that HFRT can be employed in BC patients, with non-inferiority clinical tumor control outcomes and toxicities, to shorten overall treatment course which is more convenient to the patients and cost saving to the patient and society. Of note, concomitant boost irradiation, though showed well tolerance and optimal disease control (31, 32), including hypofractionation VAMT with simultaneous boost (33), was not used in our studies after breast irradiation. Additionally, it is important to note that sequential tumor bed boost of electron was employed in this retrospective study, further suggests the safety of the HFRT.

It is worthy of discussion whether all molecular types of BC could be effectively treated with HFRT, considering that the differences in prognosis and systemic treatment are remarkable. There were few prospective randomized clinical trials focusing on the study of this topic. It seems that there was equivalent 10-year LRRFS between HFRT and CFRT groups among different molecular subtypes in a retrospective study cohort of 5,868 patients with stage I–III BC (34). Another analysis of 5,487 patients with node-positive BC reported similar 10-year LRRFS and distant recurrence-free survival between HFRT and CFRT groups, and also in high risk subgroups of grade 3, ER−/HER2−, HER2+, and N2–3 (35). Our study also showed the LRRFS and DFS were similar between HFRT and CFRT groups in locally advanced patients with HR+/HER2− and HER2+ BC, suggesting no survival benefits from CFRT under the current care of concurrent endocrine and target therapy (23, 24). However, for the extremely aggressive molecular subtype of triple negative, the application of HFRT in locally advanced disease should be evaluated with caution since these patients tended to have lower DFS in HFRT group (25% lower comparing to CFRT group) in this study, though there was no significant difference likely due to the relatively small sample size in this population. Triple negative BC is the most aggressive subtype of BC with earlier relapse and poorer survivals compared with other subtypes (36). Although the local recurrence and recurrence-free survival was reported to be similar between HFRT and CFRT groups in a large observational cohort of 538 node-negative TNBC patients (37), evidence of HFRT in locally advanced TNBC is extremely insufficient. Future study is definitely needed.

Limitations of this study include a retrospective study with imbalanced distribution of patients between HFRT and CFRT groups, and missing assessment of radiation toxicities, and limited data of longer-term survivals and relatively small sample size in high-risk patients with TNBC. Further prospective randomized trials with long-term survivals in high-risk patients with BC are needed to affirm the clinical benefits of HFRT.

## Conclusions

In this real-world study, HFRT achieved similar cumulative incidence of LRRFS and DFS in patients with BC, and in different nodal stages or in N2–3 patients with different molecular subtypes. Our study supported the application of HFRT in real-world practice to improve convenience of patients, better utilization of medical resources without sacrificing local control and survivals. This real-world study supported the routine application of HFRT in the vast majority of BC patients. However, HFRT in extremely high-risk groups (such as N2–3 patients with TNBC) should be cautious. Further prospective studies with longer-term survivals are needed in this study population to guide practice.

## Data Availability Statement

The raw data supporting the conclusions of this article will be made available by the authors, without undue reservation.

## Ethics Statement

The studies involving human participants were reviewed and approved by The University of Hong Kong-Shenzhen Hospital (# 2019 098). Written informed consent for participation was not required for this study in accordance with the national legislation and the institutional requirements.

## Author Contributions

FC: primary investigator: overall study hypothesis, study design, data collection, data analysis, result interpretation, manuscript writing and final manuscript approval. LM and PF: responsible for the statistical analysis and its various parts, results interpretation and final manuscript approval. YH, HJ, and ZX: clinic data collection and final manuscript approval. TH, EL, and YN: radiation dosimetric factors collection and final manuscript approval. AC and VH: results interpretation and final manuscript approval. F-MK: data quality control, data analysis, result interpretation, detailed manuscript preparation and final manuscript approval. All authors listed have made a substantial, direct, and intellectual contribution to the work and approved it for publication.

## Funding

This project was supported in parts by the Health Commission of Guangdong Province, China [A2021114], the Shenzhen Key Medical Discipline Construction Fund [SZXK014] and the Shenzhen Science and Technology program [KQTD20180411185028798].

## Conflict of Interest

The authors declare that the research was conducted in the absence of any commercial or financial relationships that could be construed as a potential conflict of interest.

## Publisher’s Note

All claims expressed in this article are solely those of the authors and do not necessarily represent those of their affiliated organizations, or those of the publisher, the editors and the reviewers. Any product that may be evaluated in this article, or claim that may be made by its manufacturer, is not guaranteed or endorsed by the publisher.

## Abbreviations

RT, Radiotherapy; BC, Breast cancer; CFRT, Conventional fractionated radiotherapy; HFRT, Hypofractionated radiotherapy; BCT, Breast conserving therapy; LRR, Locoregional recurrence; LRRFS, Locoregional recurrence-free survival; DFS, Disease-free survival; IMN, Internal mammary nodal; IMRT, Intensity-modulated radiotherapy; OS, Overall survival; TNBC, Triple negative breast cancer (TNBC); ER, Estrogen receptor; 2-field, 2-field tangential opposing technique; 3DCRT, Three-dimensional conformal technique; VAMT, Volume modulated arc therapy; SCF, Supraclavicular fossa; ALND, Axillary lymph node dissection; VIF, Variance inflation factor; NACT, Neoadjuvant chemotherapy; T, Tumor; N, Nodal; HR, Hormone receptor; CMF, Cyclophosphamide, methotrexate, and fluorouracil.
