# Supplementary material for: Real-World Practice of Hypofractionated Radiotherapy in Patients With Invasive Breast Cancer
Source: Front Oncol. 2022 Feb 3;12:811794. doi: 10.3389/fonc.2022.811794 (PMC8852155; doi:10.3389/fonc.2022.811794)
Supplement: Supplementary file 1 [file Table_1.docx]

**Supplementary** **Table 1: EQD2 of radiation dosimetric factors in HFRT and CFRT**

|  | Hypofractionated radiotherapy (HFRT) (n=903) | Conventionally fractionated radiotherapy (CFRT) (n=107) | P value |
| --- | --- | --- | --- |
| Mean heart dose (95% CI) EQD2—Gy | 1.4 (1.3, 1.5) | 2.8 (2.3, 3.2) | <0.05 |
| Mean dose of the total body (95% CI) EQD2—Gy | 2.8 (2.7, 2.9) | 4.3 (4.1, 4.6) | <0.05 |
| Mean dose of ipsilateral lung (95% CI) EQD2—Gy | 7.3 (7.1, 7.5) | 10.8 (10.2, 11.4) | <0.05 |
| V20 of ipsilateral lung (95% CI) EQD2—Gy | 18.0 (17.5, 18.6) | 23.1 (21.6, 24.5) | <0.05 |
| V5 of ipsilateral lung (95% CI) EQD2—Gy | 46.2 (44.4, 48.1) | 60.0 (54.7, 65.4) | <0.05 |
| Mean dose of bilateral lungs (95% CI) EQD2—Gy | 3.6 (3.5, 3.7) | 5.5 (5.1, 5.9) | <0.05 |
| V20 of bilateral lungs (95% CI) EQD2—Gy | 7.7 (7.5, 7.9) | 9.9 (9.2, 10.5) | <0.05 |
| V5 of bilateral lungs (95% CI) EQD2—Gy | 21.0 (19.8, 22.1) | 30.3 (26.3, 34.2) | <0.05 |

EQD2: Equivalent Dose in 2Gy/f; HFRT: hypofractionated radiotherapy; CFRT: conventionally fractionated radiotherapy; V20 (5): relative volume receiving more than 20Gy (5Gy).
